# Hematological Malignancies and HBV Reactivation Risk: Suggestions for Clinical Management

**DOI:** 10.3390/v11090858

**Published:** 2019-09-14

**Authors:** Alessandra Zannella, Massimo Marignani, Paola Begini

**Affiliations:** 1 Digestive and Liver Diseases Department, Liver Diseases Section, AOU Sant’Andrea Hospital, 00189 Rome, Italy; alezannella@gmail.com (A.Z.); paolabegini@virgilio.it (P.B.); 2 School of Medicine and Psychology, Sapienza University, 00189 Rome, Italy

**Keywords:** reactivation, lymphoma, hematology, immunosuppressive therapy, prophylaxis, hepatitis B virus, occult/active/inactive carrier

## Abstract

It is well known that hepatitis B virus reactivation (HBVr) can occur among patients undergoing treatment for hematological malignancies (HM). The evaluation of HBVr risk in patients undergoing immunosuppressive treatments is a multidimensional process, which includes conducting an accurate clinical history and physical examination, consideration of the virological categories, of the medication chosen to treat these hematological malignancies and the degree of immunosuppression induced. Once the risk of reactivation has been defined, it is crucial to adopt adequate management strategies (should reactivation occur). The purpose of treatment is to prevent dire clinical consequences of HBVr such as acute/fulminant hepatitis, and liver failure. Treatment will be instituted according to the indications and evidence provided by current international recommendations and to prevent interruption of lifesaving anti-neoplastic treatments. In this paper, we will present the available data regarding the risk of HBVr in this special population of immunosuppressed patients and explore the relevance of effective prevention and management of this potentially life-threatening event. A computerized literature search was performed using appropriate terms to discover relevant articles. Current evidence supports the policy of universal HBV testing of patients scheduled to undergo treatment for hematological malignancies, and clinicians should be aware of the inherent risk of viral reactivation among the different virological categories and classes of immunosuppressive drugs.

## 1. Introduction—A Brief Historical Perspective on Hepatitis B Reactivation among Patients with Hematological Malignancies Treated with Chemotherapy

The event of hepatitis B virus (HBV) reactivation (HBVr) has long been known to occur among patients undergoing treatment for hematological malignancies (HM), with reports mostly coming from the literature regarding the management of lymphomas. Historically, the first descriptions regarding the modification of serologic patterns of Hepatitis B surface antigen (HBsAg) and antibody (anti-HBs) occurring in patients with myelo- and lymphoproliferative disorders undergoing chemotherapy, were first published during the mid-1970s [1,2]. In that time frame, the dire clinical consequences of HBVr and possible approaches to treatment with the resources then available, were described [2]. During the following years, additional observations regarding the clinical and virological events developing in patients with serological signs of current or previous infection with HBV undergoing treatment for HM [3,4,5,6,7,8,9,10,11,12,13,14,15] were elucidated. Although large data sets of retrospective observations had already been published [16], it was not until 1991 that the first prospective data on HBVr among patients undergoing cytotoxic treatments became available [17]. Lamivudine (LAM) was one of the first reliable antiviral nucleoside analogues found to be effective in the inhibition of HBV replication. This medication became available during the mid-1990s [18,19] and at the end of that decade it was selected as the drug of choice to manage chemotherapy induced HBVr [20]. The seminal work by Lau paved the way to the systematic prophylactic approach using LAM to prevent of HBVr [21].

This work was followed by other investigators who tested antivirals of increasing potency in the prevention of HBVr in both HBsAg carriers [22,23,24] and in patients with signs of previous infection with the hepatitis B virus (potential occult carriers) [25,26]. In later prospective trials, entecavir (ETV) supplanted LAM as the treatment of choice to prevent HBVr in HBsAg positive lymphoma patients [27]. Acknowledgement of the role of new and specific antineoplastic agents such as the anti-CD 20 agent Rituximab (RAb) in the induction of HBVr, represented a considerable step forward in the fine tuning of prevention strategies [28]. HBVr is relevant in the treatment of these patients since it has the potential to negatively impact on clinical outcomes, by reducing the chance of cure and hampering cancer treatment completion [29,30]. In addition, it has recently been suggested that HBsAg- and anti-hepatitis B core antigen antibody- (anti-HBc) positive status, even in the absence of HBVr, might interfere with progression free survival in patients with HM [31]. HBVr among special populations has become a relevant issue, addressed in both general HBV guidelines [32,33], and in position papers dedicated to its management in specific groups of immunosuppressed patients [34,35,36,37,38]. In this paper we will focus on, critically present, and discuss the available data regarding HBVr among patients with HM.

## 2. Materials and Methods

To collect the most relevant and updated information on HBVr, a computerized literature MEDLINE search was performed using several combinations of the following terms: HBsAg, reactivation, lymphoma, hematology, immunosoppressive therapy, anti-HBc, occult carrier, and selecting potentially relevant related articles. Articles published only in abstract form were excluded. The search was performed on papers published beginning in 2015, the date of publication of the American Gastroenterological Association (AGA) position paper on HBVr [35], since it constitutes a milestone greatly contributing to the development of a systematic approach in this field. Papers published before 2015 were selected and cited when deemed relevant to the authors for their contribution on HBVr. These articles include the Italian Society for the Study of the Liver (AISF) Guidelines published in 2007 [34], and its subsequent 2017 update published online (Italian version only) [37]. These guidelines provide a systematic approach to immunosuppressed patients with serological signs of current or previous infection with HBV. This information was clearly summarized and translated in pragmatic management indications.

The results of the present search will be arranged to follow the ideal philosophy of this latter paper, critically presenting the recent literature data in order to provide practical management indications on HBVr to the clinicians involved in the care of this special subgroup of immunosuppressed patients, as specialty care providers such as hematologists and oncologists, and their consultants (infectious disease specialists, internists, gastroenterologists, and hepatologists).

## 3. Definitions

For an in-depth discussion of virological categories, and definitions of virological and clinical events in this subgroup of immunosuppressed patients, please refer to the dedicated articles of this special issue and to the following references [33,34,35,37]. A short outline of the definitions relevant to the comprehension of the present text are however provided in the following subsections.

### 3.1. Virological Categories and Phases of HBV Infection

The events following an encounter of the HBV virus with immunocompetent individuals have been schematically divided into five phases, not necessarily sequential, based on the presence/absence of hepatitis B envelope antigen (HBeAg), HBVDNA and serum transaminases (alanine aminotransferase, ALT) concentrations, and the presence/absence of parenchimal damage. These are summarized in Table 1 [33]. A more articulate nomenclature based on the description of the two main characteristics of chronicity (infection vs. hepatitis) has been proposed in 2017 [33], but it has still not been widely adopted [33]. This is also listed in Table 1 and correlated with the older terminology.

In the discussion of HBVr, we will focus on the virological categories of active carriers (AC), potential occult carriers (pOBI), and inactive carriers (IC). Briefly, AC are HBsAg positive patients characterized by the presence of liver tissue histological inflammation mediated by the antiviral immune system, and by elevated HBVDNA levels (≥2000 IU/mL). These patients can be either HBeAg or anti-hepatitis B envelope antibody (anti-HBe) positive (respectively, wild type and HBeAg minus viral variants).

The pOBI are individuals in whom the immune system controlled the HBV infection but did not provide its full clearance. After clearing HBsAg, a substantial number of these individuals still harbor viral DNA integrated in the hepatocytes and as covalently closed circular-(ccc-) DNA, which remains under the effective replicative control of the immune system. Thus, the action of immunosuppressive drugs weakening the immune system control might lead to HBVr in these individuals. These patients only show serological signs of previous viral exposure (i.e., presence of anti-HBc), very low (<200 IU/mL) or absent circulating HBVDNA, positive or negative anti-HBs [39]. A rarer form of pOBI is the occasional individual with isolated anti-HBs and no other serological HBV markers. Especially in the absence of a personal history of HBV vaccination, these individuals should be considered as possible pOBI, and in the preliminary testing phase submitted to HBVDNA testing after accurate evaluation by an expert in liver diseases. This interesting and luckily rare occurrence is probably due to mutations in the surface antigen, which renders the virus less prone to the neutralizing effect of the protective anti-HB antibodies. When undergoing profound immunosuppression, these individuals should be managed as pOBI patients [33,37,40].

The IC are HBsAg and anti-HBe positive patients with undetectable or <2000 IU/mL HBVDNA levels, even if in some individuals HBVDNA may fluctuate between 2000 and 20,000IU/mL. Their definition includes the concurrent presence of persistently normal levels of serum transaminases, no signs of histological damage or presence of minimal HBV-induced hepatic necroinflammatory activity and low fibrosis, and a clinically benign course, with a low risk of progression to cirrhosis/hepatocellular carcinoma. Progression to chronic active hepatitis B may occur. The IC state is generally characterized by the stability of these parameters during the course of extended (12-month) observation period [41]. However, this lengthy observation period can be difficult in settings requiring a rapid categorization, as in the case of patients who need to promptly undergo immunosuppressive treatments. When prolonged observation is not an option, to obtain a fast and reliable categorization of IC, quantitative HBsAg (qHBsAg) testing, HBVDNA cut-offs, liver ultrasound and fibroelastometry are also used [42,43,44]. A qHBsAg <1000 IU/mL associated with a HBVDNA <2000 IU/mL, in a one-time (spot) evaluation discriminates IC from patients from AC with sufficient diagnostic accuracy (among genotypes D, B and C) [27]. Ultrasound and fibroelastometry provide further support in staging liver disease and in differentiating AC from IC, since the former can exhibit (direct/indirect) signs of chronic liver disease and portal hypertension, while the latter measures liver stiffness (expressed in kilo pascal, KPa). Stiffness values <6 KPa virtually exclude the presence of significant liver fibrosis [37]. These modalities better differentiate AC from IC in the European and Mediterranean areas, where HBV genotype D and the HBeAg minus variant of chronic viral hepatitis B, characterized by periods of low HBVDNA levels in the presence of significant liver damage, prevail. In these same geographical areas, the occasional IC with higher HBVDNA levels (>2000–≤20,000 IU mL) described above can also be encountered, making these adjunctive diagnostic tools very helpful in reaching a precise virological classification [37].

### 3.2. Virological and Clinical Events

HBVr has been variably defined and a homogeneus consensus has not been reached, but for consistency we endorse the definitions provided by the AGA, AISF, and European Association for the Study of the Liver (EASL) guidelines and position papers [33,35,37]. In HBsAg positive individuals, HBVr it is said to occur when significant virological events develop either in the form of de novo detection of viremia in a previously negative individual (IC), or when a ≥1 log10 increase in HBVDNA is detected compared to baseline levels (obtained before starting immunosuppressive therapy), as in the case of AC. This cutoff is also used to indicate virological breakthrough in patients under antiviral treatment/prophylaxis in whom viral replication resumes. In pOBI individuals, reactivation is defined by the reappearance of serum HBsAg (reverse seroconversion) [33,35,37]. If the virological event of HBVr goes undetected, the significant clinical event of hepatitis with its burden of potentially severe complications might ensue. Progression to fulminant hepatitis and death are in fact well known potential ominous outcomes of HBVr. The event of hepatitis, regardless of clinical event severity, is defined by an increase of serum transaminases (ALT) above normal values [37] or, according to others, when a 2–3-fold rise above baseline levels or a predetermined multiple of the upper normal limit develops [45].

### 3.3. Grading Reactivation Risk according to Immunosuppressive Drug

Risk of reactivation HBVr according to immunosuppressive drug has been graded following the indications provided by the AGA guidelines as low, moderate, or high if the anticipated reactivation rate was respectively <1%, ≥1–<10%, or ≥10% [35]. According to this seminal paper, sub group classification was based on the knowledge imparted from a review of all studies used in the analysis performed by guidelines’ authors.

### 3.4. HBVr Pharmacological Management Lexicon

When deciding to either manage patients at risk for HBVr or reactivation itself, antiviral medications will be used [37] (see chapter 6 for management protocols details).

Three drug-based strategies are recognized:

(1) Treatment: administering antivirals to patients harboring an actively replicating virus.

(2) Preemptive treatment: introducing antivirals during immunosuppression if reactivation is detected as part of a monitoring protocol or if hepatitis develops.

(3) Prophylaxis: administering antivirals at baseline to patients recognized to be at a significant risk of developing reactivation as defined by their virological and clinical characteristics and according to grade of drug-induced risk of immunosuppression.

## 4. Considerations on the Issue of HBV Testing among Patients Treated for HM

Although the issue of HBVr has received increasing attention, we however witness every year the publication of case reports regarding missed diagnoses and their serious clinical implications in various settings of medicine when profoundly immunosuppressive medications are used—e.g., in the fields of oncology, immunorheumatology, and dermatology. It seems that despite the fact that the current literature on HBVr is becoming more solid and more prospective data is available, awareness surrounding HBVr is not strong enough to become part of widespread common good clinical practice. A recent paper including patients with solid and hematological malignancies, showed that substantial portions of HBV (and hepatitis C virus) infections present at the time of cancer diagnosis were unknown to patients, and that many had no known risk factors for infection, suggesting that risk-based models for screening, proposed by some [38], may be insufficient [46]. Data obtained in a survey targeting specialist treating patients with lymphoma showed however that this subgroup of physicians have a higher level of awareness concerning HBV reactivation under immunosuppression, fitting with the reported observation that a predominantly hematological practice is significantly associated with an increased likelihood of screening for HBV [47]. Accordingly, a high percentage of these professionals adopt the strategy of universal screening (>90%) [48].

Given the limited knowledge of HBV prevalence among HM in western world patients, considering that the available data mostly come from the treatment of lymphomas [49,50], even the use of screening might be insufficient. A screening procedure should be aimed at a selected population at higher risk of developing a disease condition, based on the prevalence of that condition in the population or in a selected group. Given the paucity of prevalence data among HM other than lymphoma, considering that these are mostly coming from far east populations, a preferable approach could be represented by the strategy of testing. This issue has been discussed during the recent conference on the management of HBV positive immunocompromised patients, held in Turin under the auspices of the Italian Association for the study of the liver in December 2017 [37]. As opposite to screening, testing instead applies to the single individual, and it is based on the intrinsic probability that a certain drug (i.e., rituximab, RAb) has to induce reactivation (Tommaso Stroffolini, oral presentation during the 13th AISF Special Conference on the Management of HBV-Positive Immunocompromised Patients, 15 December 2017).

We suggest that universal baseline HBV testing based on the evaluation of HBsAg, anti-HBc and anti-HBs (followed by a sensitive HBVDNA test if any of the previous returns positive). As previously stated, anti-HBs positive patients without any other serologic marker of HBV who will undergo significant immunosuppression for HM, especially in the absence of a personal history of HBV vaccination, should be considered as possible pOBI, and in the preliminary testing phase submitted to HBVDNA testing after accurate evaluation by an expert in liver diseases. Universal baseline testing should be the policy of choice when managing patients scheduled to undergo immunosuppressive treatments for HM, as also indicated by the 2017 EASL guidelines with an evidence level I and a grade 1 of recommendation according to Grading of Recommendations Assessment Development and Evaluation (GRADE) system [33]. The 2015 AGA specified that screening should be performed in those who will undergo immunosuppressive treatments at moderate or high risk of HBVr, grading the recommendation as strong and based on moderate-quality evidences according to GRADE system [35].

## 5. Factors Predisposing to HBVr

Multiple factors predisposing to HBVr among patients with serological signs of either current or previous, resolved hepatitis B infection affected by HM and undergoing immunosuppressive treatments have been identified and will be presented in this section.

HBsAg-positive patients undergoing treatment for HM run a high risk of HBVr (24–88%, median 50%). This is further increased by the use of immunochemotherapy, with most of the data coming from the RAb combination with cyclophosphamide, doxorubicin, vincristine, and prednisone (CHOP) protocol [28,51,52]. In addition, also patients with serological signs of past resolved exposure to HBV (pOBI patients) receiving immunochemotherapy, are at a high risk of reactivation (≥10%; median 16.9%; range 13.1–21.9) [24,25,52,53,54,55,56].

Several risk factors predisposing to HBVr have been identified in the different virological categories. HBVDNA concentrations do in fact correlate with HBVr risk. HBsAg positive subjects with high baseline levels of HBVDNA before starting immunosuppression (AC) are the most prone to develop HBVr as compared to those with low/undetectable viral DNA (IC) [57,58,59]. In particular, risk of HBV reactivation is 5- to 8-fold higher among HBsAg positive patients (as compared to pOBI) [60], and highest in HBeAg-positive individuals [57]. The lowest risk is present among pOBI, expecially in the absence of detectable HBVDNA [45]. Older age and male sex have also been suggested as potential risk factors for HBVr in this special population [30].

However, HM are also at a higher risk for HBVr as compared to other diseases treated with immunosuppressant drugs [61], and different HM might display different risks for HBVr [45]. Nevertheless, there are no available studies comparing similar (chemo)therapeutic regimens in different hematological settings [30]. Data on lymphoma (diffuse large cell lymphoma in particular) seem to indicate that this disease group has highest risk HBVr as compared to other HM. However, this observation might be biased since most data on HBVr in HM came from lymphoma studies [62]. In addition, it should be taken into account that it is still not clear if this observation reflects an intrinsic propensity of lymphomas in favoring HBVr or a mere effect of the medications used to treat these conditions. A long-postulated association between HBV infection and non-Hodgkin lymphoma development is well known [63], but a clear connection between this relationship and HBVr has not been found.

Multiple myeloma patients are also at risk of HBVr. In the advanced stages of this disease, a more critical immune dysregulation occurs and might predispose to HBVr [64], with reported rates of around 5% [65]. A substantial risk of viral reactivation has also been described among HBsAg patients treated for acute myeloid leukemia, with HBV-related hepatitis occurring in 8.3 per 100 person-years, and HBVr rates as high as 28% [66]. As far as pOBI patients are concerned, a recent review reported a risk of HBVr ranging from 2 to 20% among adult T-cell leukemia-lymphoma, chronic lymphocytic leukemia, and multiple myeloma [50].

Data from prospective studies conducted in pOBI patients consistently demonstrate that the presence of a significant concentration anti-HBs antibodies identifies a group at a lower risk to develop HBVr [55,67]. Nevertheless, the strategy to perform serial anti-HBs level testing to predict HBVr has not been shown to be either practical or useful [55]. Several authors suggested a differential approach based on the presence/concentration of anti-HBs antibodies in choosing prophylaxis over preemptive therapy for these two subgroups of pOBI patients, especially when dealing with those undergoing treatment with anti-CD20 drugs [55,62,67]. In a metanalysis involving 1672 hematological patients not undergoing antiviral prophylaxis, HBVr risk was shown to be 14% (95% CI: 9.4–19%) in pOBI as compared to the 5.0% (95% CI: 3.0–7.0%) observed among those also showing the additional presence of anti-HBs antibodies [68]. It was thus suggested that these pOBI patients without anti-HBs should receive antiviral prophylaxis and that anti-HBs testing can help stratify reactivation risk. However, these data also indicate that reactivation risk it is not eliminated by the presence of anti-HBs; in fact, when RAb-based treatments were used, a moderate reactivation risk (5%) remained among anti-HBs positive. Quantification of HBV core antibodies might also help in the future to predict HBVr in the group of pOBI (±anti-HBs) patients undergoing lymphoma treatment [69]. Nevertheless, results regarding this test are still preliminary and more data are needed to add this tool to the managing palette of these complex and demanding patients. Thus, from a practical standpoint, considering the lack of studies using anti-HBs titers to guide whether or not to start prophylaxis even in anti-CD-20 treated pOBI patients, we presently suggest against the use of this parameter to define the need of antiviral prophylaxis [34,35].

It is now well accepted that different classes of immunosuppressive drugs are associated with different risks of inducing HBVr. Several older drugs are notoriously known to directly act on specific promoters to induce HBV reactivation, such as corticosteroid [70] and antracyclines [71]. In general, drugs used in the treatment of HM are characterized by a severe immunosuppressive effect as in the case of RAb, the anti-CD20 monoclonal antibody par excellence, a potent B-cell depleting agent [72], well recognized to increase the chance of HBVr by more than fivefold [28]. The median rate of HBVr inherent to these agents is roughly 16.9% among pOBI, with a range of reported seroreversion to HBsAg positive status around 20–40%. Characteristically, using this drug HBVr can develop as a delayed event, again underlining its relevant and prolonged influence on the recovery of immune competence [25,28,35]. Data from recent trials suggest that the newly available anti-CD-20 obinutuzumab also induces HBVr rates not dissimilar from those occurring in RAb-treated patients [62]. Accordingly, the other currently available anti-CD20 monoclonal antibody, ofatumumab, is subject to similar precautions of use [73]. Other monoclonal antibodies also share HBVr risk such as the anti-CD52 alemtuzumab, (used to treat refractory chronic lymphocytic leukemia), mogamulizumab (for T-cell lymphoma), and brentuximab vedotin (for refractory/relapsed Hodgkin lymphoma) [30,74]. Several other newly introduced drugs, comprising biological and targeted medications also used to treat HM such as lenalidomide, bendamustine, imatinib, dasatinib, bortezomib, carfilzomib, romidepsin, temsirolimus, and phosphatidylinositol 3-kinase (PI3K) inhibitors, and BCL2 inhibitors, by acting with diverse mechanisms and on different pathways, are potentially able to interfere with the immune system and cases of HBVr have been reported. Thus, drug agencies’ warning reports regarding single drugs have been published and specific preventive managing strategies are suggested [30,75]. Also, other newly introduced therapies for the treatment of HM carry a potential risk of inducing HBVr, and even if specific reports of reactivation are missing, preventive managing strategies should be considered [76]. The introduction of drugs fulfilling the definition of “biosimilar”, i.e., a biological medicine highly similar to another biological medicine (the so-called “reference medicine”) should be considered to potentially equal the original medication in terms of HBVr risk [77,78].

HBVr also correlates with the presence of complex quasi-species, detected by ultra-deep sequencing techniques, characterized by the presence of HBsAg mutations in immune-active regions and additional N-linked glycosylation sites that might contribute to HBV escape evading both neutralizing and diagnostic antibodies [40]. These mutants might then go undetected and HBVr unnoticed by common monitoring strategies, during and after immunosuppressive treatments. A specific immune-escape mutation has recently been detected, whose presence significantly correlates with the use of RAb, suggesting that the grade of immunosuppression mediated by powerful drugs, such as the anti-CD-20 group, might progressively weaken the humoral response and favor the emergence of mutant species capable to evade antibodies [79].

In summary, even if evidence is accumulating in the recognition of potential risk factors and strategies to handle HBVr, it is evident that most of this data emerged from experiences developed on lymphoma patients, currently neither allowing the description of specific patterns of reactivation nor to give distinct management indications for the other individual subgroups of HM. To provide practical indications to clinicians dealing with the management HM and HBVr, we believe that the available data on viral reactivation among different virological classes and the understanding of the immunosuppressive action of different anticancer treatments, allow the application of the same managing strategies developed for the most studied HM to those in which hard data on the management of HBVr are lacking, at least as a temporary precaution.

## 6. Management of Patients with HM and Serological Signs of Current or Previous, Resolved Contact with HBV

As previously discussed, if the baseline testing for HBV infection of HM patients who will undergo immunosuppressive therapy has returned positive results, the evaluation of HBVr risk will proceed as a multidimensional approach, preferably involving in the decision process a specialist in liver diseases [37]. This approach includes conducting an accurate clinical and physical history, considering items such as HBV vaccination status, virological category, knowledge of the medication chosen for the treatment of the underlying HM, and the inherent grade of immunosuppression associated with both. As indicated in the definitions section, liver ultrasound and elastography are strongly recommended to complete the characterization of baseline liver status, since the outcome of HBVr will result in the worst consequences in patients with advanced liver fibrosis/cirrhosis [37]. Patients will then follow differentiated management pathways according to their HBVr risk.

AC patients who will undergo immunosuppressive treatments for HM [33] must be treated with high potency and high genetic barrier third generation anti-HBV nucleos(t)ide analogues (NUCs) such as ETV, tenofovir (TDF) or tenofovir alafenamide (TAF) as immunocompetent individuals [33,37].

IC at a high reactivation risk (≥10%) [35] should instead be managed by administration of prophylaxis with third generation NUCSs [30], which have been shown to be superior to LAM in this setting. By choosing potent and high genetic barrier medications over LAM, we better protect these frail patients, impacting over relevant clinical outcomes, preventing the development of resistant mutants, and reducing the rate of lifesaving chemotherapeutic protocol interruption [27].

Prophylaxis with LAM is also indicated to prevent HBVr among pOBI at high risk of reactivation (≥10%) and/or with detectable HBVDNA at baseline; this strategy should ideally be applied 2–4 weeks before immunochemotherapy initiation if time and clinical needs allow [34,53,54,78,80]. Recent studies conducted in Italy added further evidence to recommend the use of prophylaxis among pOBI patients at high risk of HBVr [81,82]. The use of third generation antivirals in pOBI is on the contrary questionable and debated. Recent prospective data from China, suggest that this strategy might not even be cost effective, especially in patients with low HBVr risk such as those in whom anti-HBs antibodies are present, and that a preemptive approach based on virological surveillance might thus prevent unnecessary prophylaxis in a large percentage of patients of this subgroup (96.8%) [83]. The latest 2017 EASL guidelines regarding the management of HBV infection indicate that all HBsAg-positive patients (AC, IC) should receive third-generation NUCs as treatment or prophylaxis (evidence level II-2, grade of recommendation 1), while HBsAg-negative/anti-HBc positive subjects (pOBI) should receive anti-HBV prophylaxis if at high risk of HBV reactivation (evidence level II-2, grade of recommendation1) according to GRADE system [33]. In these patients under antiviral treatment, virological response should be tested as in immunocompetent patients, in the case of both treated AC and in prophylaxed, high risk IC subjects by monitoring HBVDNA and transaminases every 6 to 12 months. In case of virological breakthrough (increase of ≥1 log10) while on treatment with ETV, either TFV or TAF will be used as rescue strategies. Similarly, TFV (TAF) will be introduced in patients previously receiving ETV or LAM. In case of partial virological control, a multidrug combo (ETV+TFV/TAF) should be considered [37]. Among LAM-prophylaxed high risk pOBI, monitoring should be performed by testing HBsAg or HBVDNA. It is still not completely clear which of the two tests has the better performance in the clinical management of these patients. Even if HBVDNA testing is more sensitive in detecting significant replication resumption, it is clinically less specific, more costly, less available, and with a longer turnaround time as compared to HBsAg testing [37]. Available data suggest that in case of a HBVr risk ≥10%, both IC and pOBI patients should undergo prophylaxis extension with the antiviral drug of choice for at least 18 months after anticancer treatment [37]. Stopping prophylaxis should only be considered if clinical remission of the underlying HM has been reached, and if no further immunosuppressive strategy is planned. After stopping prophylaxis, monitoring of serum markers (HBsAg or HBVDNA depending on the virological category) is recommended in the 6–12 months to follow [23,28].

**Figure 1:**
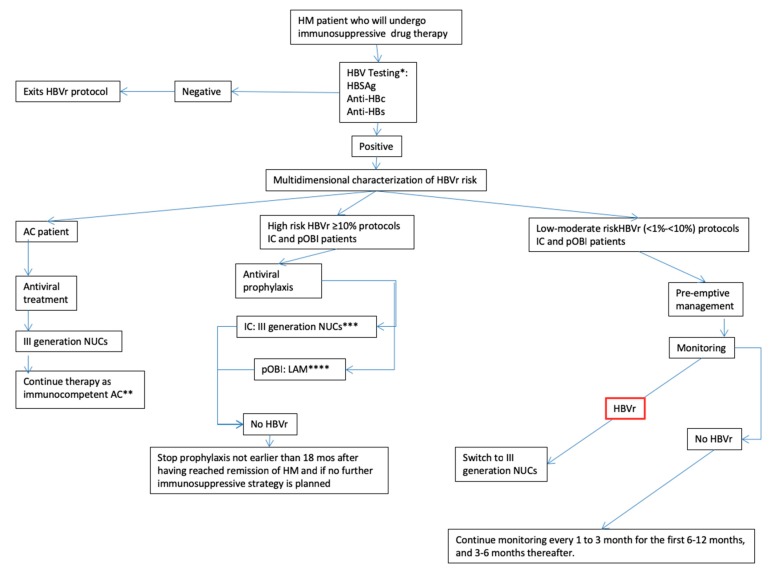
HBV-related reactivation risk: Simplified algorithm for the management of patients who will undergo immunosuppressive treatment for HM. *: Followed by sensitive HBVDNA testing if any of the previous returns positive. In case of patients only positive to anti-HBs, these should undergo accurate vaccinal medical history evaluation to define the need of HBVDNA testing. **: Might consider antiviral multidrug combo in case of partial virological contol. ***: In case of emergence of virological resistence and loss of control over viral replication, treat with alternative III generation NUC. ****: In case of emergence of viral resistence and loss of control over viral replication, switch to III generation NUC; III NUC: third generation anti-HBV nucleos(t)ide analogue; HBVr: Hepatitis B virus reactivation.

In pOBI and IC patients at a low-moderate risk of HBVr, these should be monitored for resumption of viral replication and managed by a pre-emptive strategy. If during monitoring HBVr is identified or, in the worst-case scenario, acute HBV hepatitis develops, treatment with third generation antivirals without delays is mandatory [37]. Also, HBVDNA testing should be pursued if serum transaminases increase, to test the rare, but possible, event of reactivation of mutated HBV surface antigens not detectable by all commercially available tests [40]. Unfortunately, timing and modality of monitoring strategies regulating the pre-emptive approach are mainly based on expert opinions and a knowledge gap does exist. Nevertheless, we suggest adherence to the suggestions provided by the AISF position paper. It is suggested that in patients managed pre-emptively, monitoring with ether HBsAg or HBVDNA, and ALT should be repeated every 1 (early phases of immunosuppression) to 3 months (later phases) for the first 6–12 months, and 3–6 months thereafter. No indication on when to stop monitoring after immunosuppressive treatment has been concluded is available. Nevertheless, since very late reactivation has been rarely described (up to 60 months after stopping immunosuppressive therapy [37]), it is suggested to consider this potential evidence during the follow up for HM. A simplified graphic representation of the algorithm for the management of patients who will undergo immunosuppressive treatment for HM is presented in Figure 1.

## 7. Conclusions

HBVr still constitutes a potential threat to patients undergoing treatment with potentially curative—but strongly immunosuppressive—drugs for the diseases affecting them [37]. It is our commitment as physicians to promote HBV testing before the initiation of such therapeutic protocols in patients who will undergo treatment for HM. with the goal of manage those with serological signs of current or past contact with the HBV infection, in order to prevent complications that might impair the success of their anticancer treatments.

## Figures and Tables

**Table 1:** Main characteristics of HBV infection virological categories, a simplified summary.

	2017 Nomenclature	Quantitative Serum HBsAg (IU/mL)	Serum HBeAg	Serum HBVDNA (IU/mL)	Serum Transaminases	Liver Histology	Previous Nomenclature
Phase 1	Chronic Infection	≥1000	Positive	>2000	Normal	Normal/minimal	Immune tolerant
Phase 2	Chronic Hepatitis	≥1000	Positive	>2000	Elevated	Chronic damage	Active Carrier (AC)
Phase 3	Chronic Infection *	<1000	Negative	≤2000/negative	Normal	Normal/***	Inactive carrier (IC) *
Phase 4	Chronic Hepatitis	>1000	Negative	>2000	Elevated/Fluctuating **	Chronic damage	Active Carrier (AC)
Phase 5	HBsAg-negative phase ****	Negative	Negative	<200/negative	Normal	Normal ***	Resolved acute infection/Potential occult carrier (pOBI) ****

*: In some patients HBVDNA may fluctuate between 2000 and 20,000 IU/mL and be accompanied by persistently normal ALT and only minimal hepatic necroinflammatory activity and low fibrosis. At a low risk of progression to cirrhosis/hepatocellular carcinoma if remain in this phase. Progression to chronic hepatitis may occur. **: Can intermittently be elevated; ***: If no other cause of liver diesase coexist; ****: Individuals characterized by negative serum HBsAg and positive serum hepatitis B core antigen antibodies (anti-HBc), with or without detectable serum antibodies to HBsAg (anti-HBs). Also harboring viral DNA integrated in the hepatocytes and as covalently closed circular-DNA.
